# Stearoyl-CoA Desaturase Regulates Angiogenesis and Energy Metabolism in Ischemic Cardiomyocytes

**DOI:** 10.3390/ijms231810459

**Published:** 2022-09-09

**Authors:** Ana-Maria Gan, Zuzanna Tracz-Gaszewska, Aleksandra Ellert-Miklaszewska, Viktor O. Navrulin, James M. Ntambi, Pawel Dobrzyn

**Affiliations:** 1 Laboratory of Molecular Medical Biochemistry, Nencki Institute of Experimental Biology of Polish Academy of Sciences, 02-093 Warsaw, Poland; 2 Laboratory of Molecular Neurobiology, Nencki Institute of Experimental Biology of Polish Academy of Sciences, 02-093 Warsaw, Poland; 3 Departments of Biochemistry and Nutritional Sciences, University of Wisconsin-Madison, Madison, WI 53706, USA

**Keywords:** heart, cytokines, hypoxia, fatty acids, substrate utilization

## Abstract

New blood vessel formation is a key component of the cardiac repair process after myocardial infarction (MI). Hypoxia following MI is a major driver of angiogenesis in the myocardium. Hypoxia-inducible factor 1α (HIF1α) is the key regulator of proangiogenic signaling. The present study found that stearoyl-CoA desaturase (SCD) significantly contributed to the induction of angiogenesis in the hypoxic myocardium independently of HIF1α expression. The pharmacological inhibition of SCD activity in HL-1 cardiomyocytes and SCD knockout in an animal model disturbed the expression and secretion of proangiogenic factors including vascular endothelial growth factor-A, proinflammatory cytokines (interleukin-1β, interleukin-6, tumor necrosis factor α, monocyte chemoattractant protein-1, and Rantes), metalloproteinase-9, and platelet-derived growth factor in ischemic cardiomyocytes. These disturbances affected the proangiogenic potential of ischemic cardiomyocytes after SCD depletion. Together with the most abundant SCD1 isoform, the heart-specific SCD4 isoform emerged as an important regulator of new blood vessel formation in the murine post-MI myocardium. We also provide evidence that SCD shapes energy metabolism of the ischemic heart by maintaining the shift from fatty acids to glucose as the substrate that is used for adenosine triphosphate production. Furthermore, we propose that the regulation of the proangiogenic properties of hypoxic cardiomyocytes by key modulators of metabolic signaling such as adenosine monophosphate kinase, protein kinase B (AKT), and peroxisome-proliferator-activated receptor-γ coactivator 1α/peroxisome proliferator-activated receptor α depends on SCD to some extent. Thus, our results reveal a novel mechanism that links SCD to cardiac repair processes after MI.

## 1. Introduction

Angiogenesis that occurs following myocardial infraction (MI) leads to an increase in reperfusion of the damaged myocardium by mitigation hypoxia. Interestingly, hypoxia itself is a potent angiogenic stimulus that is controlled by the activation of hypoxic signaling pathways through the stabilization of hypoxia-inducible factor 1α (HIF1α; [1]). Mice that constitutively express HIF1α in cardiomyocytes exhibit improvements in cardiac function after MI, associated with an increase in vascular endothelial growth factor (VEGF) expression and angiogenesis in the myocardium [2].

Angiogenic growth factors are released after MI, one of which is platelet-derived growth factor BB (PDGF-BB), which is needed to stabilize new blood vessels that are formed post-MI, which increases perfusion in the ischemic heart. Proinflammatory cytokines are also upregulated in the vasculature in the ischemic myocardium and may modulate the endothelial phenotype toward an angiogenic program [3]. The elevation of cytokine expression precedes the consequent increase in local matrix metalloproteinase (MMP) activity (e.g., MMP2 and MMP9) in the infarct area and an increase in the expression of atrial natriuretic peptide (ANP) and brain natriuretic peptide (BNP) [4]. Matrix metalloproteinases can be rapidly activated within minutes of ischemia by free radicals, cytokines, and hypoxia, and they can be counter-regulated to some extent by tissue inhibitors of MMPs (TIMPs; [5]). An intimate link has been well-established between cytokines and oxidative stress. The abnormal activation of non-phagocytic NADPH oxidase in response to neurohormones and cytokine activation is also a major source of reactive oxygen species (ROS) after MI. Angiogenesis can be induced by ROS through the upregulation or activation of vascular VEGF-A and HIF1α [6]. VEGF-A can activate NADPH oxidases, leading to an increase in ROS production. This feedback loop can ultimately result in an increase in VEGF-induced angiogenesis [7].

Despite recent progress in therapeutic angiogenesis post-MI, little is known about the mechanisms that are involved in hypoxia-mediated angiogenesis. Peroxisome proliferator-activated receptors (PPARs) are ligand-activated transcription factors that regulate many metabolic pathways of lipid and carbohydrate metabolism and energy homeostasis, but they are also involved in modulating inflammatory processes [8]. Several studies have sought to explain the role of PPARα in cardiac injury repair. PPARα activation reduced myocardial infarct size and improved post-ischemic contractile recovery [9]. Duerr et al. [10] found that the cardiac-restricted overexpression of PPARα led to impairments in cardiac recovery after ischemia. The transcriptional coactivator PPAR-γ coactivator 1α (PGC1α), a potent metabolic sensor that is activated by nutrients and oxygen deprivation, stimulated angiogenesis by upregulating VEGF-A [11]. Surprisingly, the induction of VEGF by PGC1α was shown to not involve the canonical hypoxia response pathway or HIF1α activity [12]. Moreover, an increase in cellular levels of adenosine monophosphate (AMP), which reflects energy deprivation, induced VEGF-driven angiogenesis through the activation of the AMP kinase (AMPK; [13]).

Given the importance of lipid metabolism in angiogenesis, we hypothesized that dysregulation of the enzymatic activity of stearoyl-CoA desaturase (SCD) might be involved in the metabolic stress-induced regeneration of cardiomyocytes after an ischemic event. The main enzyme of monounsaturated fatty acid (FA) synthesis, SCD induced the reprogramming of cardiomyocyte metabolism, which may underlie its role in the regulation of cardiac function [14,15]. Four different SCDs (SCD1-4) have been described in mice, and three SCD isoforms (SCD1, SCD2, and SCD4) are expressed in the mouse heart. SCD1 deficiency or inhibition reduced the cardiac lipid content independently of the action of PPARα by decreasing lipogenesis and elevating lipolysis [16]. Loss of the SCD1 gene improved cardiac function in obese leptin-deficient *ob/ob* mice by correcting systolic and diastolic dysfunction [15]. Moreover, SCD4 mRNA levels increased in the heart in leptin-deficient *ob/ob* mice and increased in leptin-treated *ob/ob* mice, demonstrating that SCD4 is a nutritionally regulated heart-specific SCD isoform that is a target of leptin action [17]. Palmitate (16:0), one of the main substrates of SCD, was also found to alter cardiomyocyte morphology, thereby causing cellular injury [18] and inhibit endothelial angiogenesis through the dysregulation of the Hippo/Yes-associated protein 1 pathway in endothelial cells [19].

In the present study, we investigated the mechanisms that underlie the angiogenic process in the myocardium after hypoxia and the implication of SCD1 and SCD4 in heart remodeling following acute MI. The control of cellular signaling by energy substrate utilization in hypoxic cardiomyocytes was also investigated. We found that the loss of SCD1 and SCD4 expression modulated the release of angiogenic factors and subsequently suppressed endothelial angiogenesis. We also propose that SCD altered energy metabolism in the ischemic heart by maintaining the shift from FAs to glucose as the substrate that is utilized for adenosine triphosphate (ATP) production.

## 2. Results

### 2.1. SCD Regulates VEGF-A Expression in Cardiomyocytes under Hypoxic Conditions

**Figure 1:**
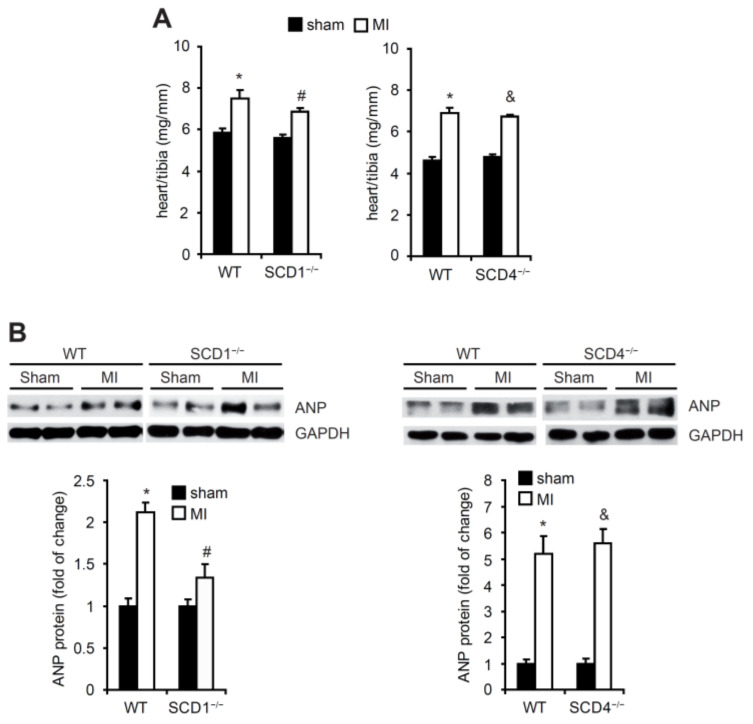
The effect of MI on the heart/tibia ratio and ANP protein levels in the heart in the WT, SCD1^−/−^, and SCD4^−/−^ mice. (**A**) The heart/tibia ratio (mg/mm) was measured to analyze cardiac hypertrophy in mice that were sacrificed 7 days post-MI. (**B**) The protein levels of ANP in the sham and post-MI myocardium were determined by Western blot. Some of the variability in ANP expression after myocardial infarction is likely related to the variable number of fibroblast cells, which infiltrated the infarct scar, therefore, in the infarcted hearts, some areas could have higher/lower the ANP content. The data are representative of *n* = 5 mice/group. The data are expressed as mean ± SD. * *p* < 0.05, vs. WT sham; ^#^ *p* < 0.05, vs. SCD1^−/−^ sham; ^&^ *p* < 0.05, vs. SCD4^−/−^ sham (two-sided *t*-test).

Seven days after the induction of MI, the mice were sacrificed. The heart/tibia ratio significantly increased in all of investigated groups (Figure 1A). The protein levels of ANP, the most relevant marker of MI, significantly increased in the heart after the MI procedure in the WT, SCD1^−/−^, and SCD4^−/−^ mice (Figure 1B).

**Figure 2:**
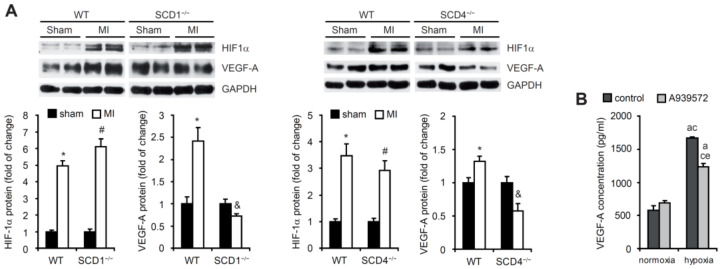
The SCD deficiency affects the HIF1α and VEGF-A expression in post-MI hearts. (**A**) Protein levels of HIF1α and VEGF-A in the sham and post-MI myocardium in the WT, SCD1^−/−^, and SCD4^−/−^ mice determined by Western blot. The data are representative of *n* = 5 mice/group. The data are expressed as the mean ± SD. * *p* < 0.05, vs. WT sham; ^#^ *p* < 0.05, vs. SCD1^−/−^ or SCD4^−/−^ sham; ^&^ *p* < 0.05, vs. SCD1^−/−^ or SCD4^−/−^ sham (two-sided *t*-test). (**B**) The HL-1 cells treated with 2 µM A939572 (SCD1 inhibitor) were subjected to hypoxia for 18 h. The VEGF-A concentration in culture medium was measured by ELISA. The data are representative of *n* = 3 independent experiments. The data are expressed as the mean ± SD. a—*p* < 0.05, vs. control/normoxia; c—*p* < 0.05, vs. A939572/normoxia; e—*p* < 0.05, vs. control/hypoxia (one-way analysis of variance [ANOVA]).

We then examined whether SCD ablation affected the protein levels of HIF1α and VEGF-A after MI. Following left coronary artery ligation, the HIF1α protein levels in the area adjacent to the infarction zone significantly increased in the WT, SCD1^−/−^, and SCD4^−/−^ mice (Figure 2A). The higher expression of VEGF-A, a HIF1α target gene, was previously shown to be observed primarily at the border zone of infarcts [2]. Our results confirmed this observation in WT mice but not in the SCD1^−/−^ or SCD4^−/−^ mice, for which we observed a significant decrease in the VEGF-A protein levels after MI compared with the sham animals (Figure 2A).

Next, we analyzed the hypoxia-induced changes in the secretion of VEGF-A in vitro by HL-1 murine cardiomyocytes. To investigate the contribution of SCD1 to these changes, we treated cells with the SCD1 inhibitor A939572. We observed higher levels of VEGF-A release to the culture medium by HL-1 cells, which were kept under hypoxic conditions (1% O_2_) compared with cells that were grown under normoxic conditions (20% O_2_; Figure 2B). The inhibition of SCD1 decreased the secretion of VEGF-A under hypoxic conditions (Figure 2B). These results suggest that SCD downregulation prevents the hypoxia-induced activation of VEGF-A expression in the ischemic heart and its secretion in HL-1 murine cardiomyocytes, independent of HIF1α.

### 2.2. SCD Regulates the Secretion of Proangiogenic Factors to Plasma in Mice Post-MI

**Figure 3:**
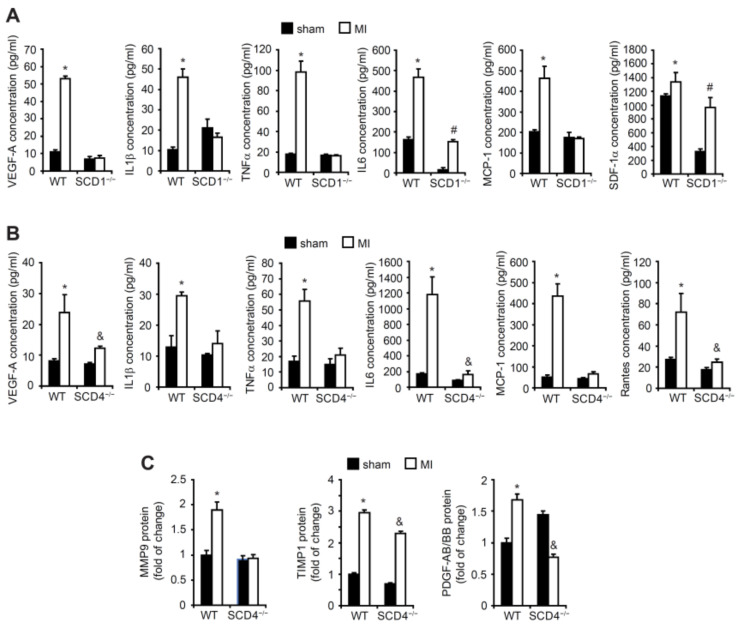
The concentrations of proangiogenic factors and inflammatory cytokines in the plasma in the post-MI SCD1^−/−^ and SCD4^−/−^ mice. The concentrations of VEGF-A, IL-1β, TNFα, IL-6, MCP-1, SDF-1a, and Rantes in the plasma of the sham and post-MI (**A**) WT and SCD1^−/−^ and (**B**) WT and SCD4^−/−^ mice were measured with the MiliplexMap Angiogenesis/Growth Factor Magnetic Bead Panel. (**C**) The content of MMP9, TIMP1, and PDGF/BB in plasma in the sham and post-MI WT and SCD4^−/−^ mice was measured with the Proteome Profiler array. The data are representative of *n* = 5 mice/group. The data are expressed as the mean ± SD. * *p* < 0.05, vs. WT sham; ^#^ *p* < 0.05, vs. SCD1^−/−^ sham; ^&^ *p* < 0.05, vs. SCD4^−/−^ sham (two-sided *t*-test).

The VEGF-A levels significantly increased in the plasma in the WT mice post-MI compared with the sham WT mice (Figure 3A,B). We compared the VEGF-A levels in plasma in the SCD1^−/−^ and SCD4^−/−^ mice post-MI to the VEGF-A levels in the plasma in the sham mice. We found that both mutant strains lost their ability to produce and secrete VEGF-A (Figure 3A,B). Inflammation emerges as one of the main stimuli of post-ischemic angiogenesis. The comparative profile of the content of inflammatory factors that were secreted to serum revealed that the level of proangiogenic cytokines (i.e., IL1-β, IL-6, TNF-α, MCP-1, SDF-1α, and Rantes) increased in serum after MI in the WT mice. In contrast, post-MI, the SCD1^−/−^ and SCD4^−/−^ mice lost their ability to secrete proangiogenic factors, with the exception of IL-6 and SDF-1α in the case of the SCD1^−/−^ mice (Figure 3A,B).

MMP9 is an important modulator of the angiogenic process in ischemic tissues. The MMP9 levels increased in the serum of the WT mice post-MI, whereas the MMP9 levels did not change in the serum in SCD4^−/−^ mice post-MI compared with the sham animals (Figure 3C). However, the levels of the anti-angiogenic cytokine TIMP-1, which exerts an inhibitory activity toward MMPs [5], were similar in the WT and SCD4^−/−^ mice post-MI (Figure 3C). PDGF signaling is involved in healing of the myocardium following infarction [20]. The PDGF family consists of four different polypeptide chains including the well-known PDGF-A and PDGF-B and recently discovered PDGF-C and PDGF-D. The disulfide-bonded homodimers PDGF-AA, PDGF-BB, PDGF-CC, and PDGF-DD and one heterodimer PDGF-AB form an active PDGF protein. Interestingly, PDGF-BB was reported to have cardioprotective properties [21]. In the present study, we measured the combined amount of PDGF-AB and PDGF-BB in the serum in the SCD4^−/−^ and WT mice post-MI. The content of PDGF-AB/PDGF-BB decreased in the serum of the SCD4^−/−^ mice post-MI compared with the WT mice (Figure 3C).

Altogether, these findings suggest that SCD may play a critical role in regulating the angiogenic process in the ischemic myocardium by stimulating the expression and secretion of proangiogenic factors that are involved actively or through dependent signaling pathways in new blood vessel formation after MI.

### 2.3. SCD Promotes New Blood Vessel Formation

**Figure 4:**
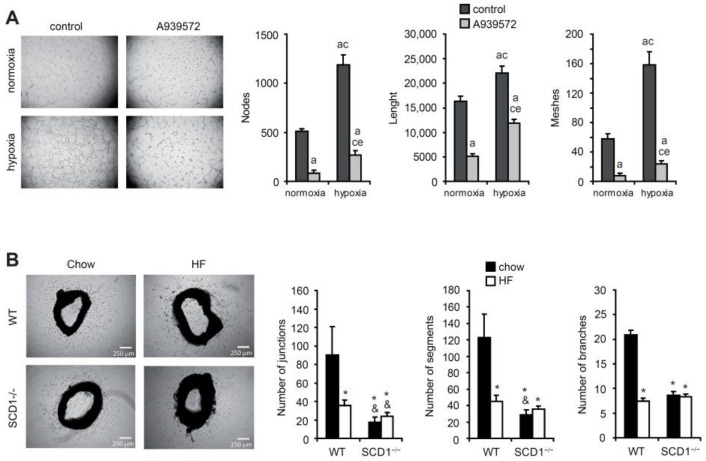
SCD1 deficiency/inhibition affects new vessel formation. (**A**) SCD1 inhibition affects the angiogenic potential of hypoxic HL-1 cardiomyocytes. Human EA.hy926 endothelial cells were cultured in conditioned medium from HL-1 cardiomyocytes that were treated with 2 µM A939572 (SCD1 inhibitor) and/or subjected to hypoxia for 18 h. The number of nodes and meshes and length of tube-like structures were measured using Angiogenesis Analyzer for ImageJ software. The data are representative of *n* = 3 independent experiments. The data are expressed as the mean ± SD. a—*p* < 0.05, vs. control/normoxia; c—*p* < 0.05, vs. A939572/normoxia; e—*p* < 0.05, vs. control/hypoxia (ANOVA). (**B**) Effect of HF diet and/or SCD1 deficiency on the angiogenic potential of aortic explants. The ex vivo ability of aorta rings to sprout blood vessel-like capillary structures was demonstrated. Photomicrographs were captured 4 days after embedding aortic rings in the collagen matrix. The number of junctions and segments and branches were analyzed using Angiogenesis Analyzer for ImageJ software. The data are representative of *n* = 3–5 independent experiments. The data are expressed as the mean ± SD. * *p* < 0.05, vs. WT chow; ^&^ *p* < 0.05, vs. WT HF (ANOVA).

Hypoxia-related new blood vessel formation follows MI to reduce heart damage. To further investigate the functional relevance of hypoxia-induced VEGF-A expression in HL-1 cardiomyocytes, a tube formation assay on Matrigel was performed. The angiogenic capacity of the factors that were released under hypoxic conditions was tested using CM from the HL-1 cardiomyocytes on the culture of EA.hy926 cells, endothelial cells that are widely used in in vitro studies of angiogenesis in cardiovascular disease. As shown in Figure 4A, the number of meshes and nodes and the total length of tube-like structures that were formed by the EA.hy926 cells significantly decreased in CM from the HL-1 cells that were treated with the SCD1 inhibitor compared with the number of these structures that formed in CM from the control cells, both under normoxic and hypoxic conditions. Thus, SCD1 inhibition in the murine cardiomyocytes suppressed the formation of endothelial tube-like structures, regardless of the oxygen concentration that was applied.

To investigate the interplay between alterations in the lipid metabolism and the angiogenic process, we next sought to establish whether an SFA-rich diet that in the long-term results in obesity and diabetes may impact the new blood vessel formation in an SCD1-dependent manner. We used an ex vivo aortic ring assay, based on the ability of aortas to sprout new vessels. Aortic rings from chow-fed WT mice possessed a higher ability to sprout vessels compared with aortas from mice that were fed an SFA-rich high-fat (HF) diet (60% calories from fat) for 6 weeks (Figure 4B). Strikingly, aortas from the SCD1^−/−^ mice lost the ability to develop capillary structures after being fed either the chow diet or HF diet compared with the chow-fed WT mice (Figure 4B).

### 2.4. SCD Affects NADPH Oxidase Subunits and MMP Expression under Hypoxic Conditions

**Figure 5:**
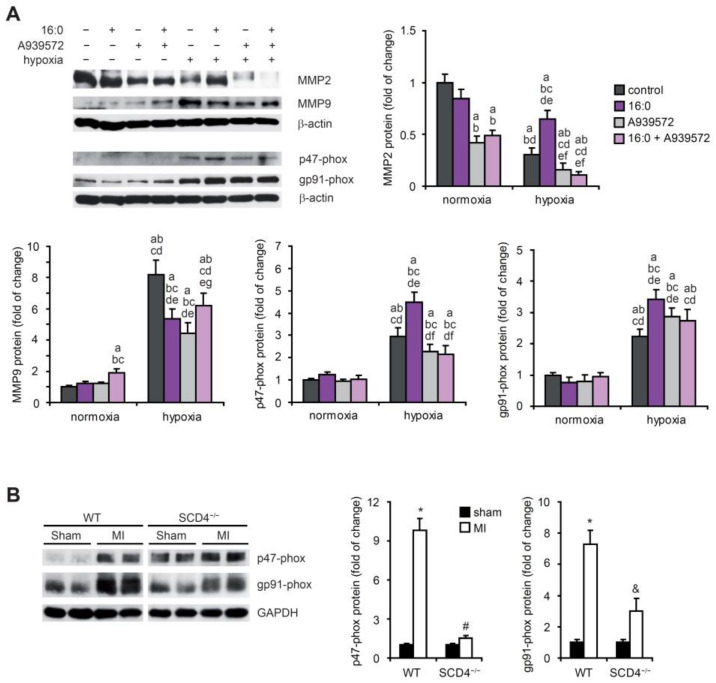
MMP2, MMP9, and NADPH oxidase subunit expression in hypoxic HL-1 cardiomyocytes and post-MI hearts. (**A**) HL-1 cells treated with 2 µM A939572 (SCD1 inhibitor) and/or 100 µM 16:0 were subjected to hypoxia for 18 h. Protein levels of MMP2, MMP9, p47-phox, and gp91-phox in cell lysates were determined by Western blot. The data are representative of *n* = 3 independent experiments. The data are expressed as the mean ± SD. a—*p* < 0.05, vs. control/normoxia; b—*p* < 0.05, vs. 16:0/normoxia; c—*p* < 0.05, vs. A939572/normoxia; d—*p* < 0.05, vs. 16:0 + A939572/normoxia; e—*p* < 0.05, vs. control/hypoxia; f—*p* < 0.05, vs. 16:0/hypoxia; g—*p* < 0.05, vs. A939572/hypoxia (ANOVA). (**B**) Protein levels of MMP2, MMP9, p47-phox, and gp91-phox in the sham and the post-MI myocardium in the WT and SCD4^−/−^ mice were determined by Western blot. The data are representative of *n* = 5 mice/group. The data are expressed as the mean ± SD. * *p* < 0.05, vs. WT sham; ^#^, ^&^ *p* < 0.05, vs. SCD4^−/−^ sham (two-sided *t*-test).

The family of MMPs has been reported to be related to cardiac pathologies, especially when the angiogenic process is involved [22]. We established that hypoxic HL-1 cardiomyocytes exhibit lower MMP2 expression and higher MMP9 expression (Figure 5A). Additional treatment with 16:0, which in an in vitro model mimics HF diet administration, caused a further increase in the MMP2 protein level but a decrease in MMP9 expression, stimulated previously by hypoxia. The SCD1 inhibitor alone or together with 16:0 decreased the MMP2 and MMP9 protein levels, which were previously increased under hypoxic conditions (Figure 5A).

Reactive oxygen species act as signaling molecules during angiogenesis. We analyzed the protein levels of two subcomponents (p47-phox and p91-phox) of NADPH oxidase (i.e., the main source of ROS production). Hypoxia stimulated the accumulation of both subunits in the HL-1 cells (Figure 5A), which likely resulted in an increase in the NADPH oxidase activity in cardiomyocytes after an ischemic event. We observed a further increase in the p47-phox and p91-phox protein levels under hypoxic conditions upon treatment with 16:0, and this increase was diminished by SCD1 inhibition in the case of p47-phox (Figure 5A). We also analyzed p47-phox and p91-phox protein levels in the heart in the WT and SCD4^−/−^ mice post-MI. The ischemic event increased the protein levels of both subunits, and the change was more pronounced in the WT mice compared with the SCD4^−/−^ mice (Figure 5B).

Our results showed that two important players in the angiogenic process, NADPH oxidase (the p47-phox and p91-phox subunits) and MMP9, were positively regulated under hypoxic conditions, whereas MMP2 was negatively regulated by hypoxia in murine cardiomyocytes. SCD1 inhibition affects the MMP2 and MMP9 expression under hypoxic conditions and p47-phox NADPH oxidase subunit expression under combined hypoxic/lipotoxic conditions. The expression of p47-phox and p91-phox was upregulated in the infarcted heart in the WT mice, and this increase was strongly attenuated by SCD4 depletion. Thus, we can conclude that SCD contributes to the ischemia-related stimulation of NADPH oxidase activity.

### 2.5. SCD1 Regulates Lipid Metabolism of Hypoxic Cardiomyocytes

**Figure 6:**
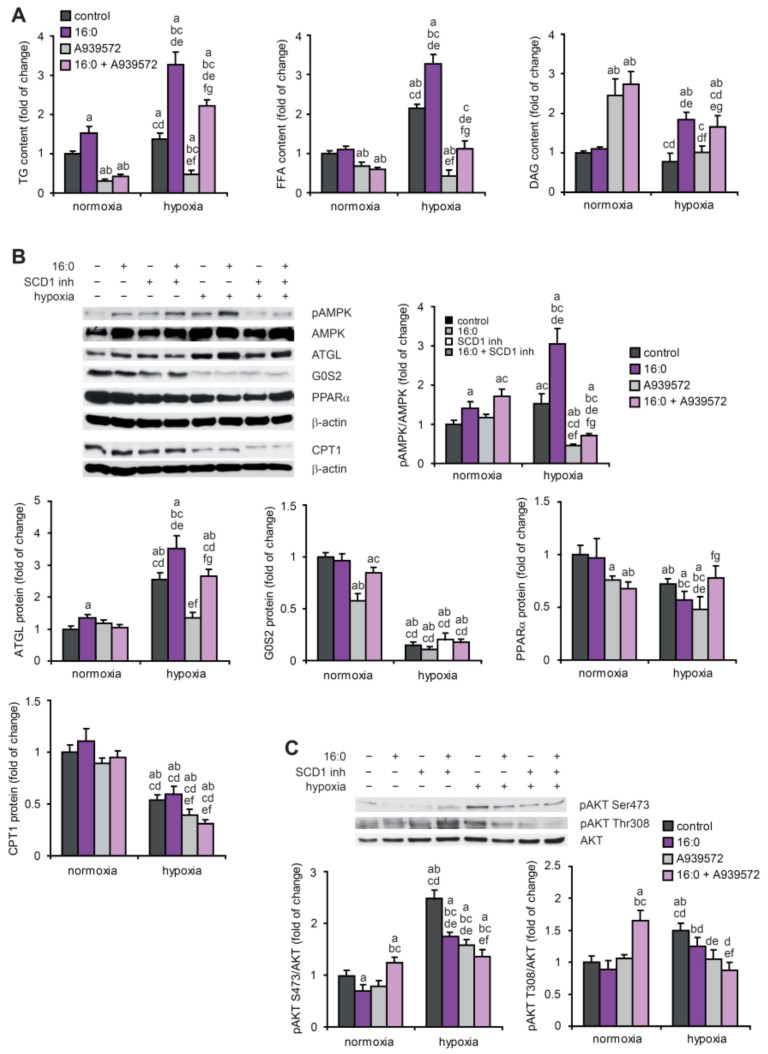
The effect of SCD1 inhibition and 16:0 treatment on lipid and energy metabolism in hypoxic HL-1 cardiomyocytes. (**A**) HL-1 cells were treated with 2 µM A939572 (SCD1 inhibitor) and/or 100 µM 16:0 and subjected to hypoxia for 18 h. Neutral lipids were extracted from the cells and separated using the thin layer chromatography method. The content of TG, FFA, and DAG was determined by densitometry. Protein levels of (**B**) pAMPK, AMPK, ATGL, G0S2, PPARα, and CPT1 and (**C**) pAKT[Ser473], pAKT[Ser308], and AKT were determined by Western blot. The data are representative of *n* = 3 independent experiments. The data are expressed as the mean ± SD. a—*p* < 0.05, vs. control/normoxia; b—*p* < 0.05, vs. 16:0/normoxia; c—*p* < 0.05, vs. A939572/normoxia; d—*p* < 0.05, vs. 16:0 + A939572/normoxia; e—*p* < 0.05, vs. control/hypoxia; f—*p* < 0.05, vs. 16:0/hypoxia; g—*p* < 0.05, vs. A939572/hypoxia (ANOVA).

To investigate the complex interplay between alterations of lipid metabolism and the angiogenic process, we mimicked the ischemic event in vitro and simultaneously applied lipotoxic conditions. We subjected HL-1 cardiomyocytes to hypoxic conditions and/or 16:0. The analysis of neutral lipid content in the hypoxic HL-1 cardiomyocytes revealed that lower oxygen availability promoted triglyceride (TG) and free FA (FFA) accumulation, and 16:0 treatment caused an additional increase in the levels of these lipids (Figure 6A). In the case of TG and FFAs, the inhibition of SCD1 also reversed these effects under normoxic conditions. Interestingly, SCD1 inhibition increased the diacylglycerol (DAG) content under normoxic but not hypoxic conditions (Figure 6A).

The growth of blood vessels is regulated by a wide range of metabolic sensors and regulators such as AMPK, PGC1α, protein kinase LKB1, sirtuin 1, and forkhead box proteins (FOXOs), particularly under nutrient deprivation conditions. For example, an increase in the cellular levels of AMP, which reflects energy deprivation, induced VEGF-driven angiogenesis through the activation of AMPK [13]. We observed an increase in the phosphorylation of AMPK under hypoxic and lipotoxic conditions in the HL-1 cardiomyocytes (Figure 6B). SCD1 inhibition suppressed AMPK phosphorylation under hypoxic conditions (Figure 6B), which may be linked to poor angiogenesis after SCD1 depletion (Figure 4). Furthermore, based on the observed increase in adipose triglyceride lipase (ATGL) protein levels and a decrease in the protein levels of the ATGL inhibitor G0/G1 switch gene 2 (G0S2), we propose that hypoxia stimulates lipolysis in HL-1 cells, and this effect is strengthened by treating hypoxic cells with 16:0. Interestingly, the increase in ATGL protein levels under hypoxic conditions was diminished by SCD1 inhibition (Figure 6B). We concluded that hypoxia suppresses FA β-oxidation in HL-1 cells through a decrease in the PPARα and carnitine palmitoyltransferase 1 (CPT1) protein levels (Figure 6B).

In patients who suffer from ischemic coronary disease and heart failure, the myocardial reliance on FAs is limited by low oxygen availability. Thus, the utilization of glucose increases [23]. Therefore, the level of protein kinase B (AKT) phosphorylation that stimulates glucose uptake was evaluated in hypoxic HL-1 cardiomyocytes. The phosphorylation of AKT at Ser473 and Thr308 was increased by hypoxia, and 16:0 treatment and SCD1 inhibition decreased AKT phosphorylation in this experimental setup (Figure 6C), suggesting lower glucose utilization.

### 2.6. SCD1 and SCD4 Deletion Affects Metabolism in the Post-MI Heart

**Figure 7:**
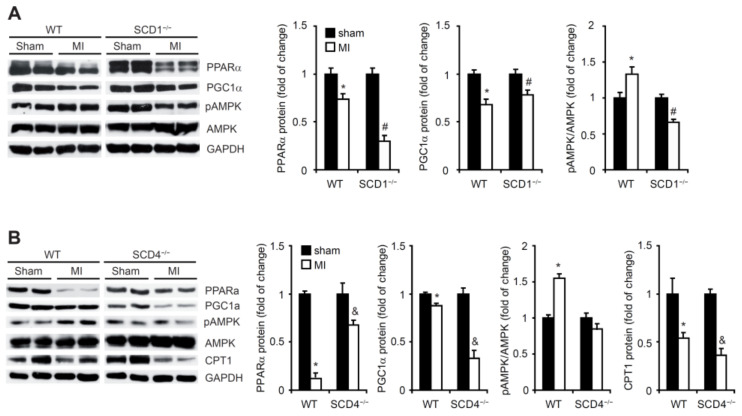
SCD1 deficiency affects fatty acid β-oxidation in post-MI myocardium. Protein levels of PPARα, PGC1α, pAMPK, AMPK, and CPT1 in the sham and post-MI hearts from the (**A**) WT and SCD1^−/−^ and (**B**) WT and SCD4^−/−^ mice were determined by Western blot. The data are representative of *n* = 5 mice/group. The data are expressed as the mean ± SD. * *p* < 0.05, vs. WT sham; ^#^ *p* < 0.05, vs. SCD1^−/−^ sham; ^&^ *p* < 0.05, vs. SCD4^−/−^ sham (two-sided *t*-test).

Based on our observations from the hypoxic HL-1 cardiomyocytes, we investigated whether SCD1 and SCD4 ablation affected lipid metabolism in the infarcted heart. PPARα has also been proposed to be one of the central regulators of cardiac metabolism during adaptation to hypoxia [24]. In the present study, MI suppressed PPARα expression in the heart in the WT, SCD1^−/−^, and SCD4^−/−^ mice (Figure 7 A,B). PGC1α, a potent metabolic sensor and regulator, binds and modulates the transcriptional activity of PPARα in the myocardium [25]. We observed a significant decrease in the PGC1α protein levels in the heart in the WT, SCD1^−/−^, and SCD4^−/−^ mice post-MI compared with the respective sham controls (Figure 7A,B).

In agreement with the HL-1 cell-based experiments, AMPK phosphorylation significantly increased in the infarcted heart in the WT mice compared with the sham mice (Figure 7A,B). However, we did not observe AMPK activation after MI in the hearts of the SCD1^−/−^ or SCD4^−/−^ mice. In the case of the SCD1^−/−^ mice, AMPK phosphorylation was even suppressed by MI. Both PPARα and PGC1α were shown to induce the expression of CPT1, although via independent gene elements [26]. In the present study, the CPT1 protein levels significantly decreased in the post-MI WT and SCD4^−/−^ hearts (Figure 7B).

## 3. Discussion

The present study demonstrated the importance of SCD1 and SCD4 in hypoxia-induced angiogenesis. We also provide evidence of the role of SCD in shaping energy metabolism in the ischemic heart. The involvement of SCD4 in cardiac function is not yet well understood, but we found that its role may overlap with SCD1 under hypoxic conditions.

In addition to their primary physiological function, cardiomyocytes are also a source of proangiogenic factors such as angiopoietin 1 and 2 and mainly VEGF-A [27]. VEGF-A plays a pivotal role in triggering the cardiac angiogenic response following acute MI [2]. Serum VEGF-A levels are positively associated with an increase in microvessel density in the infarcted area [28]. We found that MI-associated oxygen deprivation stimulated the expression of VEGF-A in murine cardiomyocytes, and VEGF-A was secreted by these cells to plasma or the culture medium. Interestingly, the post-MI increase in VEGF-A expression and secretion was abolished in the SCD1^−/−^ and SCD4^−/−^ mice and upon the inhibition of SCD1 activity by A939572 in hypoxic HL-1 cardiomyocytes. Oleic acid (18:1), the product of SCD enzymatic activity, increased the VEGF-A mRNA transcription, protein synthesis, and secretion in the rat vascular smooth muscle cells [29]. Thus, we hypothesize that the chemical inhibition of SCD1 activity in the in vitro model or loss of SCD1 or SCD4 in mice may disrupt the cardiomyocyte-derived secretion of VEGF-A by lowering the intracellular levels of 18:1. Interestingly, SCD4^−/−^ mice that underwent the MI procedure secreted more VEGF-A than the sham-treated control mice. No such effect was found in the SCD1^−/−^ mice. This indicates the possible different mechanisms that drive VEGF-A secretion in the SCD1^−/−^ and SCD4^−/−^ mice. As demonstrated in rat cardiomyocytes, mechanical stress regulates VEGF-A expression, while stretch improves the secretion [29]. However, determining whether differences in mechanical stretch affect differential VEGF-A secretion in post-MI SCD1^−/−^ and SCD4^−/−^ mice requires further detailed studies.

Oleate modulates VEGF-A expression via multiple signaling pathways that involve AKT, mammalian/mechanistic target of rapamycin, extracellular signal-regulated kinase 1/2, protein kinase Cβ, NADPH oxidase, and the mitochondrial electron transport chain complex [30]. We found that hypoxia activated AKT in an SCD1-dependent manner. We concluded that AKT-dependent signaling may be implicated in mediating the SCD1–18:1–VEGF-A proangiogenic pathway. Smith et al. [31] found that unsaturated FAs including 18:1 stimulate the expression of VEGF and other growth factors and cytokines that are involved in angiogenesis such as hepatocyte growth factor, IL-1β, IL-6, and IL-8 in mesenchymal stem cells. Therefore, a decrease in monounsaturated FA synthesis in SCD1^−/−^ and SCD4^−/−^ mice may cause a decrease in the MI-induced secretion of IL-1β, IL-6, TNFα, MCP-1, SDF-1α, and Rantes to plasma. MCP-1-induced angiogenesis is mediated by VEGF-A [32]. SCD-dependent suppression of the secretion of this angiogenic chemokine in post-MI animals may result from lower VEGF-A expression. The observed induction of VEGF-A together with proinflammatory cytokines is consistent with the findings of Maloney and Gao [33], who reported that VEGF-A mRNA was upregulated by IL-1, IL-6, IL-8, TNF-α, and transforming growth factor β in human alveolar epithelial cells. However, proinflammatory cytokines also exert proangiogenic effects independently of VEGF stimulation [34]. Altogether, we hypothesize that the observed reduction in the post-MI secretion of VEGF-A and other cytokines after SCD depletion may affect the proangiogenic potential of cardiomyocytes. The correlation between VEGF-A expression and proinflammatory cytokine production also suggests that VEGF-A may be a part of the post-MI inflammatory response, which is initiated to remove damaged cells, recruit circulating inflammatory cells to the injured myocardium, and activate reparative pathways [3]. The possible involvement of SCD in healing the infarcted myocardium is supported by the observation that SCD4 deficiency blocked the MI-associated formation of active dimers of PDGF that are involved in cardiac repair following MI [20] and contribute to the downregulation of proangiogenic processes by decreasing the VEGF production [35].

Angiogenesis is an invasive process in which the proteolytic degradation of extracellular matrix (ECM) components is required. This includes the degradation of collagen type IV by MMPs, of which MMP2 and MMP9 collagenases have been the most extensively studied [36]. In the present study, we found that the possible contribution of MMP9 to ECM degradation in the infarcted myocardium is controlled by specific SCD isoforms. Additionally, impairments in MMP9 expression and secretion upon SCD deficiency were independent of the activation of the anti-angiogenic cytokine TIMP1, which possesses an inhibitory activity toward MMPs [5]. TNFα induces MMP9 expression and activity, likely via nuclear factor κ-light-chain-enhancer of activated B cells (NF-κB), in the cells of different types [37]. The SCD-dependent stimulation of MMP9 expression that was observed in this study may result from the higher secretion of proinflammatory cytokines including TNFα upon SCD deletion. We further assumed that cardiomyocyte-derived MMP2 was not implicated in hypoxia-associated ECM degradation because its expression decreased under conditions of oxygen deficiency in the HL-1 cardiomyocytes.

The involvement of SCD1 in the process of new blood vessel formation in the in vitro model was confirmed using an ex vivo aortic ring assay, in which aortic explants that were collected from the SCD1^−/−^ mice did not exhibit the ability to sprout capillary structures. However, the role of SCD1 in regulating vascular development in normoxia and hypoxia in vivo requires further detailed studies, which is a limitation of the presented research. Next, we introduced an animal model of a HF diet that is characteristic of Western diets and leads to the development of metabolic syndrome, which increases the risk of cardiovascular diseases [38]. We found that the HF diet abolished the ability of aortic rings to generate new blood vessel-like structures. Consistent with our findings, Yu et al. [39] recently reported that a HF diet reduced the density of CD31 endothelial marker staining in the myocardial microvasculature in mice, independent of VEGF expression. The HF diet is rich in saturated FAs such as palmitic acid (16:0). Yuan et al. [19] found that 16:0 treatment inhibited human aortic endothelial cell proliferation and migration and impaired the ability of these cells to form tube-like structures, indicating impairments in angiogenesis. Chronic 16:0 treatment exerts its toxic effects on human endothelial EA.hy926 cells through impairments in the mitochondrial oxidative phosphorylation system and the intensification of FA oxidation, which leads to ROS-mediated oxidative stress and subsequent endothelial cell damage [40]. Under non-lipotoxic conditions, hypoxia induces ROS-mediated oxidative stress, which promotes the formation of new blood vessels and contributes to repair processes in the infarcted heart. For example, ROS, which are generated by NADPH oxidase, stimulate the expression and secretion of VEGF via HIF1α [41]. In the present study, the SCD4 isoform appeared to promote NADPH oxidase-associated and likely ROS-mediated oxidative stress, which further stimulated angiogenesis in the infarcted heart. Supporting this possibility, we observed SCD4-dependent decreases in the expression of the NADPH oxidase p47-phox and gp91-phox subunits and decreases in the secretion of proangiogenic factors including VEGF-A in post-MI cardiomyocytes. Thus, we hypothesize that SCD4 may regulate proangiogenic signaling in the ischemic myocardium via oxidative stress.

Myocardial ischemia is often accompanied by lipotoxicity. The expression of p47-phox and gp91-phox increased in the HL-1 cardiomyocytes upon treatment with 16:0, indicating the induction of oxidative stress in the cardiomyocytes, which is consistent with data that were reported by Joseph et al. [42]. In the case of the p47-phox regulatory subunit of NADPH, oxidase hypoxia/16:0-mediated increase in its expression was abolished by treatment with the SCD1 inhibitor, suggesting that SCD1 contributes to lipotoxicity-induced oxidative stress in hypoxic cardiomyocytes. The assembly of NADPH oxidase into an enzymatically active complex is mediated by lipid rafts. The disruption of lipid rafts decreases ROS production in an NADPH oxidase-dependent manner [43]. SCD1 inhibition impairs lipid raft formation, likely attributable to the disruption of cholesterol homeostasis [44]. The modulation of NADPH oxidase activity by SCD may occur not only through the regulation of the expression of its subunits, but also at the step of its assembly.

**Figure 8:**
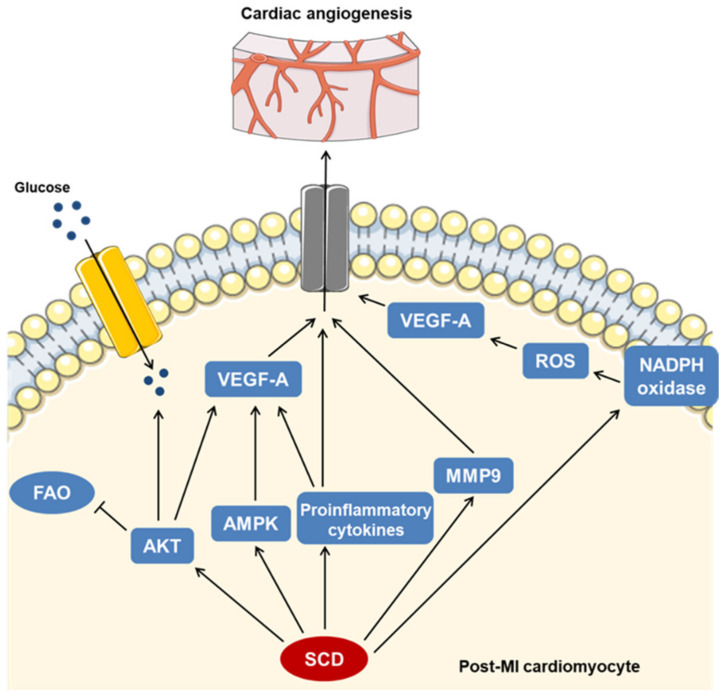
SCD-dependent signaling promotes angiogenesis in ischemic myocardium. We propose that SCD stimulates the expression and secretion of the proangiogenic factor VEGF-A through multiple cellular pathways including AKT-, AMPK-, cytokine-, and NADPH oxidase-dependent signaling. Independent of VEGF-A, SCD stimulates cardiac angiogenesis through proinflammatory cytokines and MMP9 actions. Our results indicate that SCD shapes the energy metabolism of post-MI cardiomyocytes by maintaining a shift from fatty acids to glucose as the substrate that is used for ATP production.

We found that a slight increase in TG content following hypoxia in the HL-1 cardiomyocytes was further stimulated by 16:0 treatment in an SCD1-dependent manner. However, although TG is the main form of lipid accumulation in cardiomyocytes, cardiac lipotoxicity is mostly driven by DAG and ceramides. The induction of excessive TG synthesis aims to sequester and immobilize these toxic lipid species [45]. Thus, we cannot exclude the possibility that 16:0 overload stimulates TG synthesis to protect cardiomyocytes from 16:0 lipotoxic activity. The SCD1-dependent hypoxia- and 16:0-induced increase in the FFA content corresponded to higher levels of TG, suggesting that the observed TG accumulation was accompanied by ongoing lipolysis, which appeared to result from an increase in the ATGL lipase content and a decrease in the G0S2 levels, showing an inhibitory activity toward ATGL [46]. Additionally, we concluded that SCD1 protects cardiomyocytes from the lipotoxic accumulation of DAG under normoxic conditions. The inhibition of its enzymatic activity increased the DAG content, likely through its release from TG, regardless of the 16:0 treatment. However, hypoxia abrogated the effect of SCD1 inhibition on the DAG content in cardiomyocytes, whereas higher levels of DAG upon 16:0 treatment suggest that DAG may mediate SFA-dependent lipotoxicity in the ischemic heart. Moreover, we found that the SCD1-dependent increase in FFAs that was observed upon combined hypoxia and 16:0 treatment may result from higher AMPK activity, which increases lipolysis via ATGL stimulation [47]. Considering the potential proangiogenic properties of the SCD/AMPK axis, cardiomyocyte-derived paracrine signaling that stimulates new blood vessel formation in the ischemic myocardium may be regulated by SCD1 or SCD4 via activated AMPK. The latter response promotes angiogenesis by increasing the production and secretion of nitric oxide and VEGF [48]. The possible mechanisms of the SCD-dependent regulation of angiogenesis in ischemic cardiomyocytes are presented in Figure 8.

Ischemia induces PGC1α expression in cultured skeletal myotubes or in the subcortex in the brain to counteract the devastating effects of oxygen deprivation on mitochondria [12,49]. However, consistent with our findings, a reduction in PGC1α expression is observed in the ischemic myocardium. PGC1α co-activates PPARs to induce the import and oxidative breakdown of FAs to fuel the high energy requirements of the heart [11]. Thus, we suggest that the suppression of PGC1α/PPARα signaling may reduce the rate of FA β-oxidation in the ischemic heart. This is also supported by our observation that hypoxia decreased the protein content of CPT1, which enables FA transport from the cytosol to the mitochondrial matrix.

SCD1 stimulates PI3K/AKT signaling to increase cell proliferation and survival and promote tumorigenesis [50,51]. In the present study, we observed an SCD1-dependent increase in AKT-activating phosphorylation in hypoxic HL-1 cardiomyocytes, which may induce further cellular responses to hypoxia including an increase in survival and the activation of HIF1α-dependent signaling. AKT reduces cardiac FA β-oxidation through the downregulation of PPARα and PGC1α [52]. We concluded that the observed decrease in PPARα and PGC1α expression in the post-MI heart may result from the activation of AKT, which is controlled in a top–down manner by SCD1. Accordingly, we assumed that SCD1 may stimulate glucose oxidation in hypoxic cardiomyocytes by enhancing glucose uptake via AKT. Thus, SCD appears to counteract the imbalance of cardiac energy metabolism that is caused by hypoxia by modulating AKT signaling.

In conclusion, we propose that SCD1 and SCD4 activity overlap in the activation of processes that seek to restore proper functioning of the heart after MI. In the cascade of SCD-dependent molecular pathways, energy and metabolic signaling are intertwined with the proangiogenic pathways. For example, SCD stimulates activation of the key metabolic sensors AMPK and AKT, which adjust energy metabolism to the restricted oxygen supply and also contribute to the induction of angiogenesis in the ischemic heart. The oxidative stress-dependent stimulation of new blood vessel formation may also be regulated by SCD activity. Our results reveal a novel mechanism that links SCD to cardiac repair processes after MI.

## 4. Materials and Methods

### 4.1. Materials

AMPK (catalog no. sc-25792), ANP (catalog no. sc-515701), CPT1 (catalog no. sc-31128), G0S2 (catalog no. sc-133423), MMP2 (catalog no. sc-13595), MMP9 (catalog no. sc-393859), p47-phox (catalog no. sc-74514), gp91-phox (catalog no. sc-74514), PGC1α (catalog no. sc-518025), PPARα (catalog no. sc-398394), VEGF-A (catalog no. sc-7269), and β-actin (catalog no. sc-47778) antibodies were obtained from Santa Cruz Biotechnology (Santa Cruz, CA, USA). AKT (catalog no. 2938), phosphorylated AKT at Ser473 (pAKT[Ser473]; catalog no. 9271), pAKT at Thr308 (pAKT[Thr308]; catalog no. 9275), ATGL (catalog no. 2138), and phosphorylated AMPK at Thr172 (pAMPK; catalog no. 2531) antibodies were obtained from Cell Signaling Technology (Hartsfordshire, UK). Glyceraldehyde 3-phosphate dehydrogenase (GAPDH; catalog no. MAB374) and HIF1α (catalog no. MAB5382) antibodies were obtained from Merck (Darmstadt, Germany). The other chemicals were purchased from Sigma (St. Louis, MO, USA) unless otherwise specified.

### 4.2. Animals

The generation of SCD1 knockout (SCD1^−/−^) mice was as previously described [53]. SCD4 floxed mice were generated on the C57BL/6 background at the University of Wisconsin Biotechnology Center’s Transgenic Animal Facility, similar to the SCD3 floxed mice [54]. To disrupt the SCD4 coding sequence, a homology recombination vector (HRV) that contained exon 3 flanked by LoxP sites was designed. For recombinant clone selection, the PGK-NeoR cassette was inserted into HRV and delimited by FRT sites. The construct carried the herpes simplex virus thymidine kinase cassette located on the construct 3′-terminus and separated from the NeoR cassette by a 3′-homology arm. The linearized construct was electroporated into stem cells and selected with geneticin. SCD4 floxed/floxed mice were crossed with eIIa cre+ mice to yield SCD4 global knockout mice. SCD4 floxed mice were screened for the exon 3-deficient (~750 bp product) and wildtype (WT; ~1200 bp product) locus by polymerase chain reaction.

Male WT C57/BL6 mice and SCD1^−/−^ and SCD4^−/−^ mice at 12 weeks of age were used in the study. The animals were housed in a pathogen-free facility at room temperature under a 12 h/12 h light/dark cycle with ad libitum access to water and standard chow. All of the protocols that were used in this study were approved by the First Local Ethical Committee for Animal Experiments in Warsaw.

### 4.3. Induction of Myocardial Infarction

Myocardial infarction was induced by permanent left coronary artery ligation. Briefly, the mice were anesthetized with a mixture of ketamine/xylazine (100/10 mg/kg body weight) and orotracheally intubated by cannulating the trachea with a 20-gauge blunt needle that was attached to a mouse ventilator (Ugo Basile, Italy) via a plastic Y-connector. Tidal volume and ventilation rate were adjusted according to the mouse’s weight (e.g., a ventilation rate of 133 breaths per minute and tidal volume of 0.2 mL for a 30 g mouse). The chest was shaved, and left thoracotomy was performed in the fourth intercostal space. Ligation was performed 1 mm distal from the tip of the left auricle with a 7-0 silk thread suture. After ligation, the chest cavity was closed, and the muscles and skin were separately sutured with 6-0 polypropylene sutures. Voluntary respiration of the animal was restored by gently removing the intubation needle. For analgesia, all animals were injected subcutaneously with buprenorphine hydrochloride (0.1 mg/kg) post-surgery and on the first postoperative day. Another group of mice underwent sham ligation, in which they were subjected to similar surgical procedures without tightening the suture around the coronary artery. Animals were sacrificed 7 days post-MI, and the left ventricle of the heart was excised and frozen in liquid nitrogen. MI induction in SCD1^−/−^ and SCD4^−/−^ mice were separate experiments using separate WT control groups. Although the WT mice used in both experiments were the same age (12 weeks), they had different initial body and heart weights. This variation was reflected in the calculated HW/tibia ratio between the two experiments performed.

### 4.4. Cellular Hypoxia Induction in HL-1 Cardiomyocytes

HL-1 cardiomyocytes were obtained from W.C. Claycomb (Louisiana State University, New Orleans, LA, USA). Cells were cultured on a gelatin (0.02% [*w*/*v*])/fibronectin (10 μg/mL) matrix and maintained in Claycomb medium supplemented with 10% (*v*/*v*) fetal bovine serum, 2 mM glutamine, 0.1 mM norepinephrine, 100 U/mL penicillin, and 100 U/mL streptomycin in a 5% CO_2_ atmosphere at 37 °C [55]. The medium was changed every 24 h. For the generation of normal and hypoxic HL-1 cardiomyocytes, cells were grown to high confluency under normoxic (20% O_2_) and hypoxic (1% O_2_) conditions. Cells were subjected to hypoxia for 18 h in an O_2_/CO_2_ incubator (INCO 153, Memmert, Germany) in a 1% O_2_ atmosphere achieved by air replacement with nitrogen. The culture medium was pre-equilibrated in 1% O_2_ for 24 h. Additionally, to inhibit SCD1 activity, cells were pre-incubated with 2 µM of the SCD1 inhibitor A939572 (Biofine International, Blain, WA, USA) [56,57] for 4 h under normoxic conditions and then co-supplemented with 100 µM 16:0-bovine serum albumin (BSA) conjugate for the next 18 h under hypoxic conditions. At the end of the incubation period, the conditioned medium (CM) was collected and centrifuged at 2500× *g* for 20 min at 4 °C to remove the cellular debris. Supernatants were stored at −80 °C.

### 4.5. Enzyme-Linked Immunosorbent Assay

The content of VEGF-A that was secreted by HL-1 cells was quantified in CM using an enzyme-linked immunosorbent assay (ELISA; R&D Systems, Minneapolis, MN, USA) according to the manufacturer’s instructions.

### 4.6. Determination of Angiogenic Profile

Plasma collected from sacrificed mice was used and subsequently processed according to the manufacturer’s protocols. Briefly, to analyze the content of secreted molecules in murine plasma after MI, two independent methods of quantification were used. The first method was the MiliplexMap Angiogenesis/Growth Factor Magnetic Bead Panel, which included VEGF-A, interleukin-1β (IL-1β), IL-6, tumor necrosis factor α (TNFα), monocyte chemoattractant protein-1 (MCP-1), stromal cell-derived factor-1α (SDF-1α), and Rantes detection (MAGPMAG; Merck, Germany). A Luminex analyzer (MAGPIX; Thermo Scientific, Pittsburgh, PA, USA) was used to acquire and analyze the data. The second method was the Proteome profiler system, in which array membranes were incubated with plasma and a biotinylated antibody cocktail overnight at 4 °C (Mouse Angiogenesis Array Kit, R&D Systems). After several washing steps, membranes were incubated with streptavidin conjugated to horseradish peroxidase (HRP) and then exposed to the HRP substrate. Signal intensity was analyzed with the FujiFilm Luminescent Image Analyzer LAS-3000, and pixel densities were analyzed with ImageJ software.

### 4.7. In Vitro Tube Formation Assay

Human umbilical vein EA.hy926 endothelial cells (American Type Culture Collection, Manassas, VA, USA) were cultured in Dulbecco’s modified Eagle’s medium (DMEM) with 20 mM HEPES, 100 U/mL penicillin, 100 U/mL streptomycin, and 10% fetal bovine serum (FBS). Cells were maintained in a humidified 5% CO_2_ atmosphere at 37 °C. The ability of EA.hy926 cells to form capillary-like tubes in CM from HL-1 cells was assessed by employing the Matrigel assay (Millipore, Billerica, MA, USA). The bottom of a 96-well culture plate was coated with Matrigel (50 μL per well) and incubated for 1 h at 37 °C for gelification. Gels were overlaid with 100 μL of CM, which was collected from normoxic and hypoxic HL-1 cells, and then 40,000 EA.hy926 cells were added per well. Tube formation was monitored using an AF7000 inverted phase contrast microscope, and photographs were taken with a DCF 35DFXR2 camera (Leica, Wetzlar, Germany). The number of capillary networks, branching points, meshes, and total lengths were analyzed using Angiogenesis Analyzer for ImageJ software [58].

### 4.8. Ex Vivo Mouse Aortic Ring Assays for Angiogenesis

The mouse aorta was removed from the SCD1^−/−^ and WT mice under septic conditions. The dissected aorta was transferred to a dish that contained cold Opti-MEM medium (Thermo Scientific). To avoid contamination with other cell types, excess fat tissue was quickly removed using a forceps. The aorta was cut with a scalpel into ~0.5 mm rings that were serum-starved by incubation in Opti-MEM overnight at 37 °C and 5% CO_2_, as previously recommended [59]. The next day, one aortic ring per well of a 96-well plate was embedded into a collagen matrix. After 4 days of incubation at 37 °C with 5% CO_2_, images of newly formed blood vessel-like capillary structures were captured with a Leica AF7000 microscope. The number of junctions and segments and branches were analyzed using Angiogenesis Analyzer for ImageJ software [58].

### 4.9. Western Blot Analysis

HL-1 cells were collected and lysed for 30 min in ice-cold lysis buffer that contained 20 mM Tris-HCl (pH 7.4), 150 mM NaCl, 1 mM ethylenediaminetetraacetic acid (EDTA), 1% Triton X-100, 1 mM dithiothreitol, 0.1 mM phenylmethane sulfonyl fluoride (PMSF), 1 mM sodium orthovanadate (Na_3_VO_4_), 10 μg/μL leupeptin, 5 μg/μL pepstatin A, and 2 μg/μL aprotinin. For further analyses, whole-cell lysate supernatants that were obtained by centrifugation at 12,000× *g* for 20 min at 4 °C were used. Left ventricle samples from the SCD1^−/−^, SCD4^−/−^, and WT mice were lysed in ice-cold lysis buffer that contained 20 mM Tris-HCl (pH 7.4), 2 mM ethylene glycol-bis(2-aminoethylether)-*N*,*N*,*N’*,*N’*-tetraacetic acid, 2 mM EDTA, 2 mM Na_3_VO_4_, 1 mM PMSF, 10 mM β-mercaptoethanol, 10 μg/μL leupeptin, 5 μg/μL pepstatin A, and 2 μg/μL aprotinin and centrifuged at 10,000× *g* for 20 min at 4 °C. Protein content in the lysates was determined using the Bio-Rad Protein Assay (Bio-Rad, Hercules, CA, USA) with BSA as the reference. Protein samples were separated with 10% sodium dodecyl sulfate-polyacrylamide gel electrophoresis gels and transferred to polyvinylidene difluoride membranes (Millipore, Billerica, MA, USA). Western blot analysis was performed using the appropriate antibodies. The activity of the HRP-conjugated secondary antibodies was detected using the SuperSignal West Pico PLUS Chemiluminescent Substrate (Thermo Scientific) and quantified by densitometry. Protein levels are expressed relative to the abundance of GAPDH or β-actin. Phosphorylated protein levels are expressed relative to the abundance of the unphosphorylated form of the respective protein.

### 4.10. Lipid Analysis

Lipids were extracted from HL-1 cardiomyocytes according to Bligh and Dyer [60]. Extracted lipids were separated by thin-layer chromatography on silica gel 60G plates (Merck, Darmstadt, Germany) in heptane/isopropyl ether/glacial acetic acid (60/40/4 [*v*/*v*/*v*]) with authentic standards. To visualize the lipid bands, the plate was soaked in a water solution of 10% cupric sulfate and 8% phosphoric acid and then burned in at 140 °C for 20 min. The content of the separated groups of lipids was then quantified by densitometry.

### 4.11. Statistical Analysis

The data are expressed as the mean ± SD, with *n* = 5 mice/group. The data that were obtained using HL-1 cells are representative of three independent experiments. Multiple comparisons were performed using one-way analysis of variance (ANOVA) followed by Tukey’s *post hoc* test using Prism 8.3.0 software (GraphPad, La Jolla, CA, USA). A two-sided *t*-test was applied when differences between the two groups were analyzed. The level of significance was *p* < 0.05.

## Data Availability

The data that support the findings of this study are available from the corresponding author upon reasonable request.
